# Ovulation but not milt production is inhibited in fathead minnows (*Pimephales promelas*) exposed to a reproductively inhibitory pulp mill effluent

**DOI:** 10.1186/1477-7827-12-43

**Published:** 2014-05-22

**Authors:** Andrew Waye, Wudu E Lado, Pierre H Martel, John T Arnason, Vance L Trudeau

**Affiliations:** 1 Department of Biology, University of Ottawa, 30 Marie-Curie, Ottawa, ON K1N 6N5, Canada; 2 FPInnovations, 570 Saint-Jean Blvd, Pointe-Claire, QC H9R 3J9, Canada

**Keywords:** Fathead minnow, Pulp mill effluent, Reproductive inhibition, 5-day fathead minnow spawning assay, Ovulation, Milt

## Abstract

**Background:**

A 5-day fathead minnow (FHM) spawning assay is used by industry to monitor pulp mill effluent quality, with some mill effluents capable of completely inhibiting spawning. The purpose of this report is to characterize the effect of an inhibitory effluent on egg and milt production in FHM.

**Methods:**

Eight tanks were treated with an inhibitory effluent while eight were kept with clean water. Each tank contained two males and four females as per the 5-day FHM spawning assay used by industry. Females were stripped of ovulated eggs and males of milt in four effluent-exposed and four control tanks. Eggs oviposited in every tank were also counted and checked for fertilization and data analyzed with 2-way ANOVA.

**Results:**

We show that female, but not male, fathead minnow reproductive function is impaired in the 5-day fathead minnow spawning assay used by industry to evaluate pulp mill effluent quality in Canada. Milt production was not changed in the control or exposed males mid-way and at the end of the five day exposure (p > 0.05; n = 8). Total egg production (stripped + oviposited) was impaired (p < 0.05) in fathead minnows exposed to effluent (288 eggs/tank, n = 4 tanks) compared to those in control tanks (753 eggs/tank, n = 4 tanks).

**Conclusions:**

Our results indicate that males are able to detect female signals and prepare appropriately for spawning while in females inhibition of ovulation is occurring somewhere along the hypothalamus-pituitary-gonad reproductive axis. These results suggest female-specific neuroendocrine disruption and provide mechanistic insight into an assay used by industry to assess pulp mill effluent quality.

## Background

In 1992 Canada initiated national Environmental Effect Monitoring (EEM) studies to measure the impacts of pulp and paper mill effluents (PPME) on benthic invertebrates and adult fish. In the 20 years of EEM cycles, it has been identified that there is a national trend of larger livers and smaller gonads in fish living downstream of PPME; reproductive and metabolic disruption by PPME has therefore been a major research focus for environmental toxicologists and endocrinologists. While several specific chemicals have been identified, none have been convincingly demonstrated to be responsible for the effects observed in the wild (for reviews, see [1-3]). Considerable effort has been made to identify solutions to the effects of effluent on wild populations [4] and one result of these efforts was the development of an *in vivo* 5-day fathead minnow (*Pimephales promelas*) spawning assay that is now used by industry at the laboratories of Forest Product Innovations (FPInnovations) to assess the quality of Canadian pulp and paper mill discharge and investigation of cause studies [5,6]. This assay was developed following multi-generational life-cycle studies with fathead minnows (FHM) where the effects of metabolic and reproductive disruption seen in wild fish populations exposed to effluents were successfully mimicked. During these life-cycle studies, the ability of fathead minnows to spawn was identified as a relatively quick and sensitive way to measure reproductive disruption by pulp mill effluents and the 3-week egg production assay designed by Ankley et al. [7] was adopted for monitoring effluent quality [8]. The resulting 3-week FHM spawning assay was further optimized to a 5-day assay, where active effluents are able to elicit rapid (overnight) and reversible spawning inhibition of breeding fathead minnows [5].

Our work to date on pulp mill effluents has led to the hypothesis that PPME contain neuroendocrine disruptors that inhibit reproductive processes in fish [9-13]. Since ovulation and sperm release are controlled by a neuroendocrine cascade, we hypothesized that such a rapid and reversible inhibition of spawning may be neuroendocrine in nature (for example, perhaps by disrupting the luteinizing hormone (LH) surge necessary to trigger spawning) [11]. We have since performed assays to demonstrate that effluents and wood feedstock used by mills contain potentially neuroactive chemicals that can act on the dopamine and GABA systems [9,10,12,13].

The objective of the present study was to determine whether it is the males or the females whose ability to spawn is inhibited. More specifically, we tested whether ovulation in females and/or milt production in males were affected by exposure to an inhibitory effluent. If ovulation and milt production are observed after exposure, we could refute our hypothesis that spawning inhibition is neuroendocrine in nature [9,11] and that the pituitary is not able to produce an LH surge. If an inhibition of ovulation and/or milt production is observed, then the disruption of the neuroendocrine control of reproduction hypothesis [9,11] is still valid, although other mechanisms may also be at play.

## Methods

### Pulp mill description and parameters

Effluent samples were from a thermomechanical pulp (TMP) mill in Eastern Canada that produces 889 t/d of newsprint. Effluent flow is 32 m^3^/t of pulp (Table 1). Pulp is produced using wood furnish comprised of 70-75% spruce and 25-30% fir as well as deinked pulp and is bleached with sodium hydrosulphite and hydrogen peroxide. Treatment of the effluent occurs using flotation units (15-20% of effluent flow), and secondary treatment comprises of air activated sludge in a sequential batch reactor.

**Table 1:** Pulp mill description and operating procedures

**Pulping**	**Brightening agent**	**Furnish**	**Product**	**Production**	**Water usage**	**Primary treatment**	**Secondary treatment**
TMP deinked pulp	Na_2_S_2_O_4_/H_2_O_2_	25-30% fir, 70-75% spruce	Newsprint	889 t/d	32 m^3^/t	Flotation^1^	SBR
		Deinked pulp: > 80% ONP					

^1^Treats only 15-20% of primary effluent flow in flotation units.

*TMP* thermomechanical pulp; *ONP* old newspaper; *SBR* air activated sludge in sequential batch reactor.

### Sample collection and storage

Grab samples were taken from the final effluent outflow on May 22, 2012 and shipped in two 1000 L bulk containers lined with food-grade polyethylene to FPInnovations in Pointe-Claire, Québec, for fathead minnow exposures. Upon arrival, contents were transferred to 210 L polyethylene barrels and stored at 4° Celsius. Exposures began on May 25, 2012 and were terminated on May 30, 2012. The effluent sample was analysed for pH (Orion Model 1230, Thermo Fisher Scientific, Ottawa, ON, Canada), conductivity (Orion Model 162A, Thermo Fisher Scientific, Ottawa, ON, Canada ), dissolved oxygen (YSI 52, Yellow Springs Instruments Inc., Yellow Springs, OH, USA), and ammonia (Accumet 950, Fisher Scientific, Ottawa, ON, Canada). Effluent biochemical oxygen demand, chemical oxygen demand, hardness, and total suspended solids were measured as per the American Public Health Association [14] and resin and fatty acids analysis was performed by gas chromatography [15]. Measured physicochemical parameters are described in Table 2.

**Table 2:** Measured physicochemical parameters of tested effluents

**Date sampled**	**pH**	**Conductivity**	**Hardness**	**DO**	**NH**_**3**_	**BOD**	**COD**	**SS**	**RFA**
May 22, 2012	7.2	2060 μS	918 mg/L	4.8 mg/L	0.1 mg/L	10 mg/L	187 mg/L	9.9 mg/L	0.01 mg/L

*DO* dissolved oxygen; *BOD* biological oxygen demand; *COD* chemical oxygen demand; *SS* suspended solids; *RFA* resin and fatty acids (detection limit 0.01 mg/L).

### Fathead minnow reproduction assays and effluent exposures

Fish were bred, cultured, selected, and cared for at a fathead minnow colony at the wet labs of FPInnovations (Pointe-Claire, QC, Canada) and the exposure regime occurred according to previously published methods [5]. Briefly, two males and four females were held in 12.5 L aquariums for a pre-exposure period of 7 days. Aquariums contained spawning substrates made of two 8 cm lengths of food-grade polyvinyl pipes with a 10 cm diameter cut in half longitudinally which were monitored in the mornings for daily egg production. Successful fertilization of spawned eggs was confirmed by observations using a dissection microscope. Groups exhibiting the highest reproductive performance were selected for the experiment.

Effluent exposures began on May 25, 2012 at a concentration of 100%. This concentration is the standard for industrial testing of effluents and investigation of cause studies [6]. At this concentration nearly complete spawning inhibition and no mortality was observed. Each treatment (control, 100% effluent, control stripped, and 100% effluent stripped) was performed with 4 replicate tanks. For the experiment and pre-exposure period, the replicates were kept in glass 12.5 L tanks under flow-through conditions with 4 to 6 tank volume renewals per day and were aerated at a minimum of 6.5 mL/min/L. The photoperiod used was 16 hours light and 8 hours dark. Each day tank pH (7.52 to 8.27), dissolved oxygen (>74.9%), and temperature (25°C +/- 1°C) was monitored and the spawning substrates were checked for egg production. For the stripped FHM in 100% effluent, fish were stripped just prior to effluent exposure, and on the third and last day (day five) of the experiment. All fish were sacrificed upon termination of experiment on day five. Snout to fork body length and wet body weight and gonad weight were recorded.

Fish were anaesthetized (in a solution of 100 mg/L MS-222 (Sigma-Aldrich, Toronto, ON, Canada) and females were stripped of ovulated eggs and males were stripped of milt just before the initiation of the experiment (T = 0) and at days 3 and 5 (end of exposure). To strip eggs, anaesthetized females were held gently in the hand and the abdomen was massaged lightly using strokes of the thumb towards the posterior. Eggs released from the oviduct were collected with an eye dropper, weighed, and put aside for counting. To strip milt, males were similarly held and massaged and milt was collected in Fisherbrand Micro-Hematocrit Capillary Tubes (Fisher Scientific, Pittsburg, PA, USA) by suction applied via a tube connected to the mouth and subsequently weighed. When males were stripped of milt upon completion of the experiment, milt weight was added to gonad weight to correct to unstripped male gonadal weight.

### Statistical analyses

**Figure 1:**
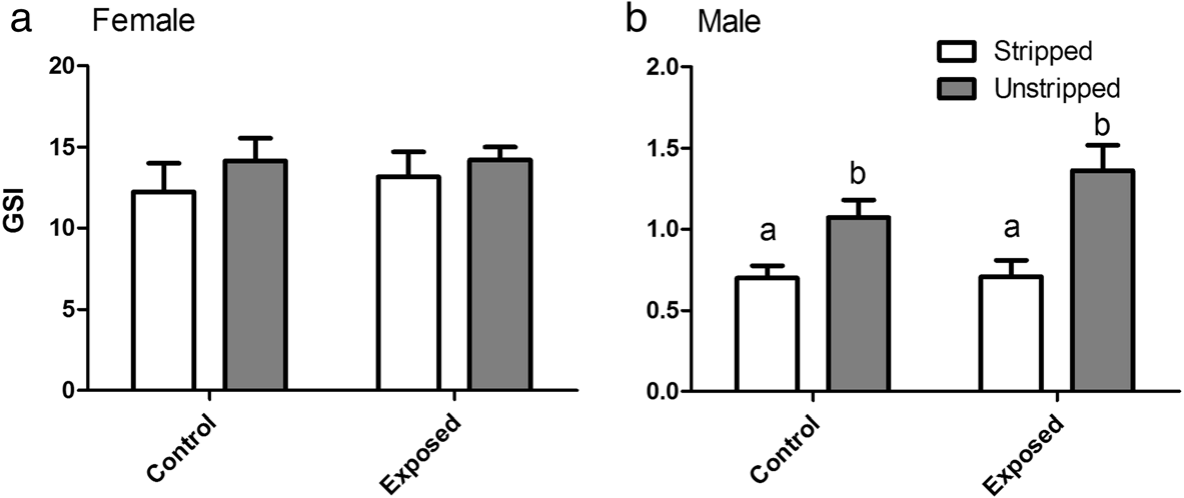
**Mean female (a) and male (b) gonadosomatic index (GSI).** The GSI of white bars (which represent control or exposed fish that were stripped of ovulated eggs or milt) was calculated by adding gonad weight and weight of stripped milt or eggs. Grey bars represent control or exposed fish that were not handled. Error bars represent 1 standard error of the mean and significant differences denoted by differing letters (p < 0.05).

**Figure 2:**
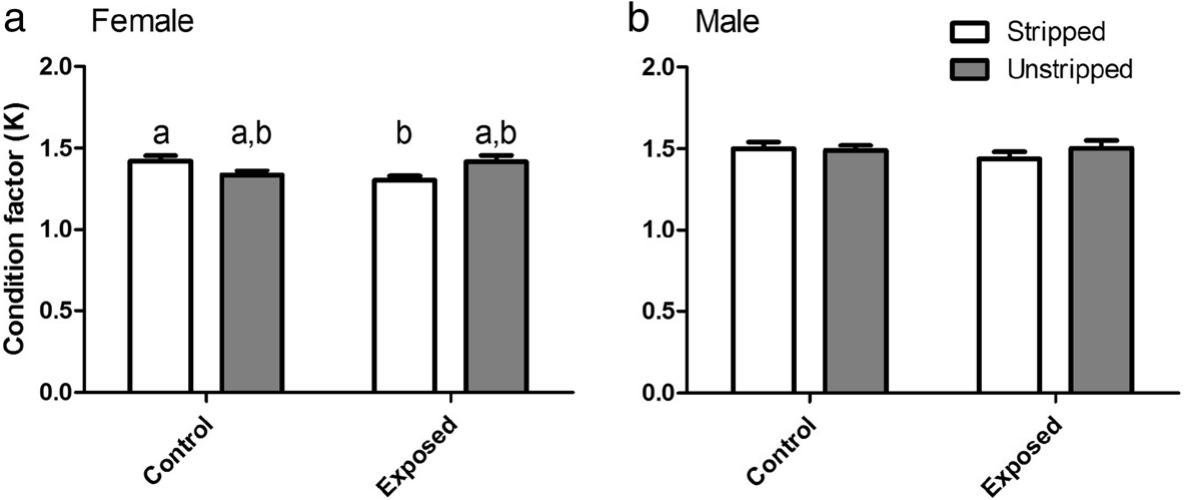
**Female (a) and male (b) condition factor (K).** White bars represent control or exposed fish that were stripped of ovulated eggs or milt while grey bars represent control or exposed fish that were not handled. Error bars represent 1 standard error of the mean and significant differences denoted by differing letters (p < 0.05).

**Figure 3:**
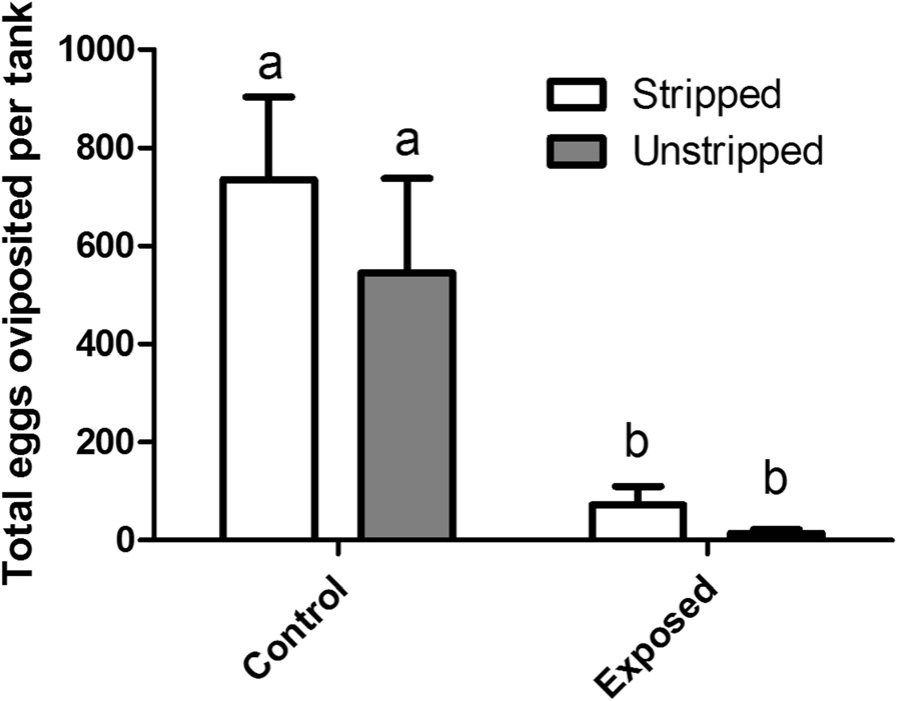
**Cumulative number of eggs oviposited per tank.** White bars represent control or exposed tanks where fish that were stripped of ovulated eggs or milt while grey bars represent control or exposed tanks where fish that were not handled. Error bars represent 1 standard error of the mean and significant differences denoted by differing letters (p < 0.05).

**Figure 4:**
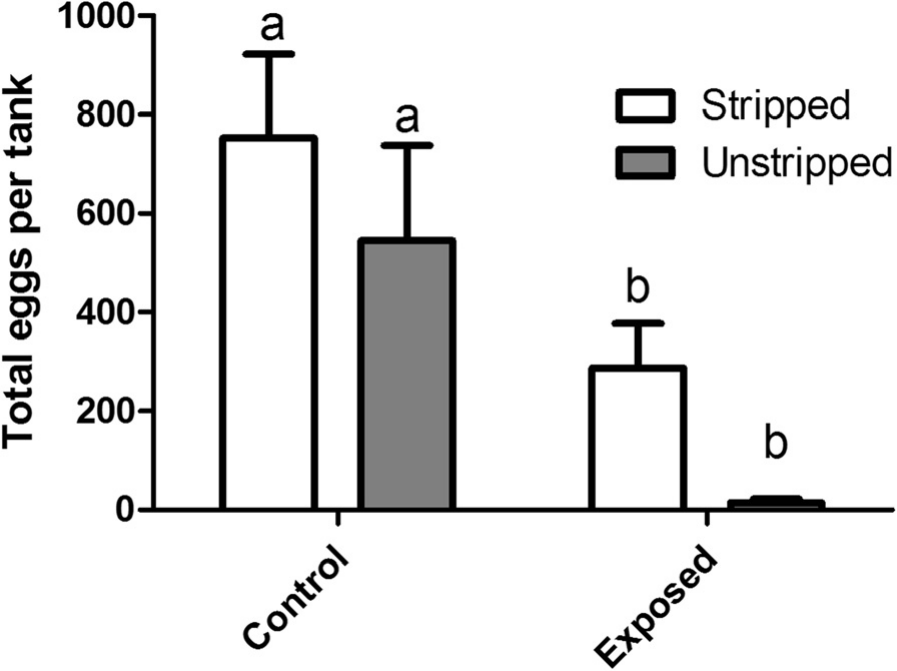
**Cumulative number of total eggs produced (stripped + oviposited) per tank.** White bars represent control or exposed tanks where fish that were stripped of ovulated eggs or milt while grey bars represent control or exposed tanks where fish that were not handled. Error bars represent 1 standard error of the mean and significant differences denoted by differing letters (p < 0.05).

**Figure 5:**
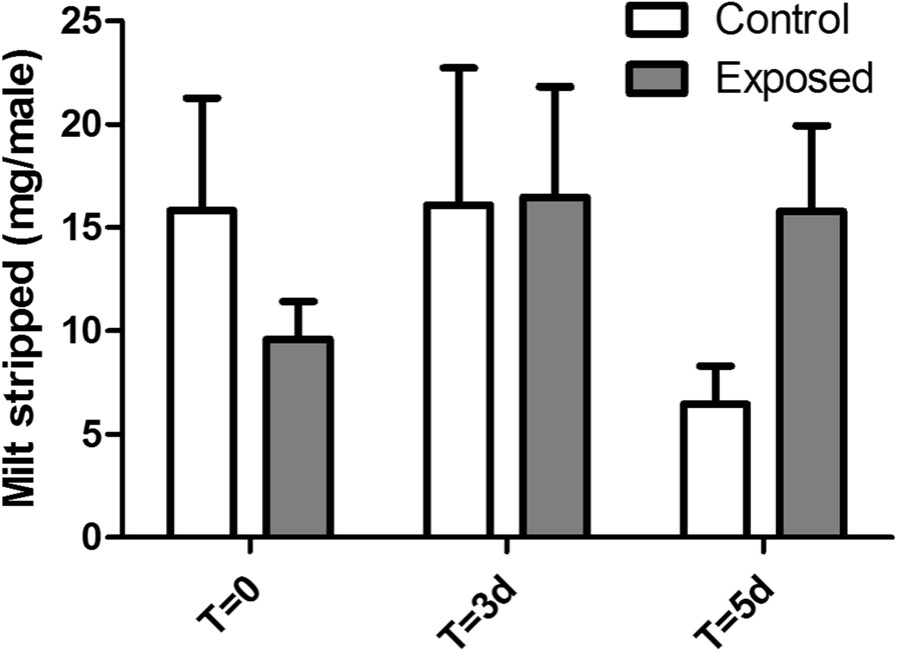
**Weight of milt stripped per male fathead minnow.** White bars represent control tanks while grey bars represent exposure to pulp mill effluent. Error bars represent 1 standard error of the mean and no significant differences are observed (p > 0.05).

Male and female length, weight, gonadosomatic index (GSI; Figure 1), condition factor (K = [wet weight(g)/fork length(cm)^3^] × 100; Figure 2), number of eggs spawned per tank (Figure 3), total eggs produced per tank (Figure 4), and male milt production (Figure 5) were analysed using two-way ANOVA. All statistics were performed using SPSS v. 17.0 (IBM, San Jose, CA, USA) and figures were created in GraphPad Prism v. 5.01 (GraphPad Software, La Jolla, CA, USA).

## Results

### Morphometric parameters

Neither effluent exposure nor stripping had an effect on mean fish fork length or weight during the course of the exposure. For GSI, an effect on milt levels was observed from stripping, resulting in lower GSI in stripped males compared to unstripped males (Figure 1b; p < 0.001). No effects were observed from effluent exposure compared to controls for both stripped and unhandled groups. No significant differences were detected for female GSI measurements Figure 1a). A statistical difference in K was detected between stripped exposed (mean K 1.42 +/- 0.06 95% C.I.) and unstripped exposed (mean K 1.30 +/- 0.05 95% C.I.) females, but not when compared to controls (Figure 2a).

### Egg production in female fathead minnows

There were no significant differences in mean cumulative egg production (p > 0.05) between pre-exposure treatment groups, and all replicates spawned successfully (i.e., there were some eggs that were successfully fertilized) over the seven day pre-exposure period and over the duration of the experimental exposure.

Exposure to the 100% TMP effluent caused a significant (p < 0.05) inhibition of spawning (Figure 3). Untreated female groups oviposited the statistically same numbers of eggs regardless of stripping as did the effluent-exposed groups (p > 0.05; Figure 3). When comparing the total numbers of eggs (oviposited + stripped), we observed that the total egg production in untreated groups is the same, regardless of stripping, as is the total egg production in the effluent exposed groups (Figure 4; p > 0.05). The difference in total egg production between unstripped control and exposed groups was significant (p < 0.05) as was the difference between the stripped control and effluent exposed groups (Figure 4). There was no significant effect of stripping the fish, nor was there a significant effect of the interaction between effluent exposure and stripping (p > 0.05).

### Milt production in male fathead minnows

There was no significant difference (p > 0.05) between the amount of milt stripped from the control males and the males exposed to 100% effluent at any of the time points where milt was sampled (at T = 0 and after days 3 and 5 of exposure; Figure 5).

## Discussion

### Morphometric parameters

We did not observe any changes in morphometric parameters during the experiment with the exception of male GSI (Figure 1b) and female K (Figure 2a). We observed the GSI in males was lower at the end of the experiment in males that had been stripped of milt during the experiment. This is expected and may be explained by an effect of handling and stripping on the males resulting in higher demands on the gonad for milt (and thus a reduced gonad weight) when compared to unstripped fish. The difference detected in female K was likely due to slightly, but not significant (p = 0.0564), longer mean female fork length in the exposed stripped tanks. Changes in morphometric parameters were not observed during the shortened 5-day fathead minnow spawning assay in the study by Kovacs (2007). In the 5-day study of 7 mechanical mill effluents tested in the 5-day FHM spawning assay, an increased gonad weight from two mill effluent exposures was the only morphometric parameter observed to change [16], while in a study of 7 Kraft mill effluents, female body weight was lower in only one treatment [17]. The authors of these studies state that these differences do not indicate effluent-related effects since measurements were taken at the end of the experiment after the fish had been removed from the effluents and kept in well water for five days [16,17]. In the longer 21-day assay, no changes in morphometric parameters were seen in males but in females mean fork-length was lower in one of the treatments (a 2% Kraft mill effluent) while mean weight and condition factor was higher in another treatment (a different Kraft mill effluent at 40% concentration) [18].

### Egg production in female fathead minnows

Fewer eggs were laid in the female groups exposed to a TMP effluent. In order to assess if the females were not ovipositing despite ovulating, we stripped ovulated eggs from the exposed females. Reasons for females ovulating but not spawning might include males not detecting the release of pre- and post-ovulatory pheromones due to pheromone binding/adsorption to effluent constituents or blockage of male olfactory receptors resulting in male failure to initiate spawning behaviour or perhaps ovulated females are able to detect non-ideal conditions for their eggs and decide to forgo spawning until conditions improve.

Our results (Figure 4) clearly demonstrate that ovulation is impaired in effluent-exposed fish compared to controls because we were unable to strip many ovulated eggs from the exposed females. If the exposed females were indeed ovulating, we would expect the total eggs per tank (oviposited + stripped) to be similar to the total eggs in the control tanks, only with a higher proportion of the total eggs being stripped compared to controls.

In teleost fish, ovulation is triggered by a surge in luteinizing hormone (LH) from the pituitary, which is under the direct stimulation of gonadotropin-releasing hormone [19]. Dopamine and GABA are very important neurotransmitters in the reproductive axis because they respectively inhibit and stimulate LH release [20-22]. This inhibitory input by dopamine is potent, such that co-injection of a dopamine antagonist with a GnRH agonist is required to induce the LH surge and spawning in teleosts [23]. *In vitro* experiments on effluent extracts by Basu et al. [9] identified that extracts of a Canadian TMP effluent contained ligands for the dopamine type-2 receptor among other neuroendocrine targets important to reproductive control. In follow-up experiments, several effluents [13] and hardwood [10] and conifer [12] feedstocks have also identified potential neuroactivities.

It is not possible to measure LH in FHM because the LH radioimmunoassay has not been developed for this species. Nevertheless, we hypothesize that LH release may be reduced such that the female FHM exposed to the PPME were unable to ovulate. Prior to our work, a study by [24] on a white sucker (*Catostomus commersoni*) population exposed to a bleached Kraft mill effluent from a mill in Terrace Bay, ON, Canada, clearly demonstrated that LH and sex steroids in exposed wild white suckers were depressed compared to those from control sites. Upon injecting females with a GnRH agonist, the size of the resulting LH surge observed in control fish was not seen in those populations exposed to effluents, nor did ovulation occur (while it did in controls). This study indicated that either the pituitary had lost GnRH sensitivity (due to lesions or perhaps other mechanisms) or that other inhibitory signals, such as dopamine, might be suppressing LH release. Due to the inhibitory dopaminergic tone, for example, it is necessary to co-inject dopamine receptor antagonists with GnRH to stimulate spawning in many fish species.

It is also possible that while an LH surge is indeed occurring in the exposed females, the ovaries of these individuals may not be responding appropriately, but this is speculative because we did not directly measure steroid product. *In vitro* studies (Gibbons et al. [25]; McMaster et al. [26]) have demonstrated an impaired induction of steroidogenesis in the gonadal tissue of fish exposed to pulp mill effluents: both testosterone and 17β-estradiol production was inhibited in ovarian follicles stimulated by human chorionic gonadotropin in tissues collected from wild white sucker (*Catostomus commersonii*) downstream of a sulphite pulp mill [26], and forskolin-stimulated 17β-estradiol production was inhibited in ovarian follicles collected from trout-perch (*Percopsis omiscomaycus*) downstream of a thermomechanical/de-inked pulp mill [25]. However, the significance of inhibited steroid production is unclear since previous studies have demonstrated that reduced spawning rates in fish exposed to effluents do not appear to be associated with several steroidogenic endpoints [27].

Additional mechanisms of action of pulp mill effluents that may help explain the results seen in our study. Pharmacological inhibition of steroidogenic brain aromatase or 3β-hydrosteroid dehydrogenase/Δ^5^-Δ^4^ isomerase (3β-HSD) was also able to elicit rapid inhibition of spawning in fathead minnows. The aromatase inhibitor fadrozole results in decreased brain aromatase activity in fathead minnows and in these fish, rapid spawning inhibition and impairment of oocyte maturation was observed [28]. Plasma estradiol and vitellogenin concentrations were also decreased in these females exposed to fadrozole. Inhibition of 3β-HSD by trilostane also caused a rapid inhibition of spawning in fathead minnows as well as decreased levels of vitellogenin [29], so the observed effects in our study could be explained by effects at the level of the gonad or liver in addition to effects on brain. However, many effluents are strongly anti-reproductive, but inconsistently affect steroidal pathways [30,31]. It is possible that effluents are inducing a stress response and that increased cortisol levels play a part in the inhibition of ovulation, but there is little evidence for stress induction as measured by cortisol in fish [2,32]. Regardless of the hormonal response, pulp effluents are acting somewhere in the hypothalamus-pituitary-gonad axis and it is more than likely that this includes effects at all three levels.

### Milt production in male fathead minnows

We observed no effects from exposure to a TMP effluent that inhibits spawning on male milt production (Figure 5), indicating that the reproductive inhibition is a female rather than a male effect. Males are likely detecting sex pheromones that are released by females to signal to males that they have ovulated, since the males are producing milt.

Typically, in female cyprinid fish the LH surge is followed shortly thereafter by the release of a pre-ovulatory hormone (17α,20β-P or an analog) from the somatic follicle cells in the ovary, which immediately precedes ovulation. This pre-ovulatory hormone is released to the environment as a sex pheromone and is detected by males. Upon detection, the males also experience an increase in LH and within hours milt volume begins to increase (for review, see [33]). While we conclude that this TMP effluent inhibits ovulation in female fathead minnows, it is evident that a few eggs are still ovulated and oviposited (Figures 3 and 4). On day 3 of the experiment, we were able to strip eggs from 8 of the 16 females in the effluent treatment and 0 of 16 females in the control tanks. On day 5, we were again able to strip eggs from 8 of the 16 exposed females, and only from 4 of the control females. Presumably, any ovulated eggs in the control tanks had already been oviposited, explaining the difference in egg stripping success rate between the control and exposed tanks. Since some females were still able to ovulate a few eggs in the effluent-treated tanks, despite a dramatic decrease in oviposition (Figure 3), it seems that there may be sufficient pre-ovulatory hormone released to the water to stimulate the appropriate milt production in males.

## Conclusions

We show that females exposed to an inhibitory TMP effluent in the 5-day fathead minnow spawning assay prevented ovulation in females, but not milt production in males. This result has several implications into the mechanistic effects of inhibitory effluents in this assay, with future avenues of research focussing on inhibitory neuroendocrine pathways in the reproductive axis or on the ability of the gonad and pituitary to detect and respond to signals that originate in these pathways. Furthermore, milt production in males was not different in males exposed to this effluent when compared to controls, indicating that males likely remain sensitive to female reproductive pheromonal signaling.

Sex-specific effects on brain function were observed in fathead minnows exposed to five inhibitory secondary-treated effluents in the 5-day fathead minnow spawning assay [34]. Urotensin 1 and RevErbβ2 mRNA levels were preferentially affected in females compared to males. Only one effluent decreased urotensin 1 mRNA in male hypothalamus, while all five effluents caused a decrease in females. In the telencephalon, one of the effluents caused an increase in urotensin 1 mRNA levels in females, while no changes were observed in males. For RevErbβ2, four of the five effluents resulted in different mRNA levels in the hypothalamus than controls (three decreased, one increased), while in males, only one of the five effluents caused a decrease in relative mRNA expression. RevErbβ2 is a nuclear receptor that plays an important role in circadian rhythms [35]. Since photoperiod is a strong determinant of reproductive success in fathead minnow [36], a shift in RevErbβ2 expression in the brain may be a mechanism by which spawning is disrupted. In another study, sex-specific effects were also in fathead minnow liver gene expression in fish exposed to secondary treated pulp mill effluents [37]. While no changes in gene expression was observed in female livers, males had increased expression of androgen receptor, estrogen receptor β, and cytochrome P4501A, although these effluents did not inhibit spawning during a 6-day exposure at 25% v/v effluent concentration [37].

We conclude that there is a female-specific reproductive effect of exposure to a thermomechanical pulp mill effluent. There was the rapid inhibition of ovulation without any major effects on milt production in males. The fathead minnow continues to be an amenable model for ecotoxicology and endocrine disruption research, and future focus on the mechanism behind the female-specific effect observed in this study could allow for the development of faster and more cost-effective assays by which to investigate environmental contaminants and their sex-specific reproductive effects. In this way, effective mitigations strategies may also be developed.

## Competing interests

The authors declare that they have no competing interests.

## Authors’ contributions

AW, WEL, PHM, VLT, JTA collaborated in developing experimental and sampling methodology. AW and WEL collected samples/data, while AW, VLT, and JTA performed data analysis. AW wrote the paper and all authors read, provided input, and approved the final manuscript.
